# The Effects of Continuous Positive Airway Pressure Therapy for Secondary Cardiovascular Prevention in Patients with Obstructive Sleep Apnoea: A Systematic Review and Meta-Analysis

**DOI:** 10.31083/j.rcm2306195

**Published:** 2022-05-27

**Authors:** Huan Li, Yueyang Pan, Yake Lou, Yujie Zhang, Leran Yin, John E Sanderson, Fang Fang

**Affiliations:** ^1^Department of Sleep Medical Center, Beijing Anzhen Hospital, Capital Medical University and Beijing Institute of Heart, Lung and Blood Vessel Diseases, 100029 Beijing, China; ^2^Princeton International School of Mathematics and Science, Princeton, NJ 08540, USA; ^3^Department of Cardiology, The Second Affiliated Hospital of Chongqing Medical University, 400010 Chongqing, China; ^4^Department of Sleep Medical Center, Beijing Anzhen Hospital, Capital Medical University, 100029 Beijing, China; ^5^Beijing Anzhen Hospital, Capital Medical University and Beijing Institute of Heart Lung and Blood Vessel Diseases, 100029 Beijing, China

**Keywords:** continuous positive airway pressure, obstructive sleep apnea, major adverse cardiovascular or cerebral events, secondary prevention

## Abstract

**Background:**

Obstructive sleep apnoea (OSA) is highly prevalent and significantly associated with major adverse cardiovascular events (MACEs). Continuous positive airway pressure (CPAP) treatment has a protective effect on cardiovascular events in OSA patients. However, whether CPAP therapy significant reduces the risk of recurrent cardiovascular (CV) events in OSA patients with established cardiovascular or cerebrovascular diseases remains disputed. We aim to evaluate the effect of CPAP on recurrent cardiovascular outcomes in moderate to severe OSA patients with previous cardiovascular or cerebrovascular diseases.

**Methods:**

We searched the electronic databases (PubMed, EMBASE, and Cochrane library) from their inception to August, 2021. Only randomized controlled trials (RCTs) that described the association of CPAP treatment in patients with cardiovascular or cerebrovascular disease and OSA were included in our analysis. The primary outcome of interest was major adverse cardiac or cerebral events (MACCEs), a composite endpoint of myocardial infraction (MI), non-fatal stroke, CV mortality; secondary outcomes included all-caused death, cardiac mortality, myocardial infraction, atrial fibrillation, heart failure, repeat revascularization, angina, stroke, and transient ischemic attack. In addition, subgroup analyses based on CPAP adherence were performed.

**Result:**

Six RCTs of 4493 participants were included in the analysis. Compared with usual care, CPAP therapy did not significantly reduce the risk of recurrent MACCEs odds ratio (OR) 0.94, 95% confidence interval (CI) 0.79–1.12, *p* = 0.5, CV mortality (OR 0.83, 95% CI [0.54–1.26], *p* = 0.37), myocardial infarction (OR 1.09, 95% CI [0.8–1.47], *p* = 0.6), heart failure (OR 0.94, 95% CI [0.66–1.33], *p * = 0.71), stroke (OR 0.9, 95% CI [0.67–1.23], *p* = 0.52), or all-cause death (OR 0.86, 95% CI [0.63–1.16], *p* = 0.32). However, the subgroup analyses revealed that CPAP can decrease the risk of CV mortality (OR 0.25, 95% CI [0.08–0.77], *p* = 0.02) and stoke (OR 0.39, 95% CI [0.15–0.97], *p* = 0.04) in patients who used it more than 4 hours.

**Conclusions:**

CPAP therapy was not associated with reduce the risk of MACCEs in OSA patients with a history of chronic cardiovascular disease who utilize CPAP <4 hours/night, although CPAP appeared to have a positive effect on CV mortality and stroke among those who used CPAP >4 hours. The correlation between CPAP and the prognosis of OSA patients warrants further study.

## 1. Introduction

Obstructive sleep apnea (OSA) is characterized with recurring episodes of partial or complete collapse of the upper airway during sleep, resulting in fragmented sleep, intermittent hypoxia, intrathoracic negative pressure, and other pathological physiological processes which are especially associated with cardiovascular complications [1], such as hypertension [2], myocardial ischemia, heart failure [3], arrhythmia, stroke [4], and even sudden cardiac death [5]. The prevalence of OSA in adults in the general population ranges from 9% to 38% [6], and even higher in an aging population, what’s more, the incidence of OSA varying from 20% to 60% in the people over 65 years and increased to 78% in women and 90% in men aged 70 to 85 years [7]. In addition, previous study has demonstrated that OSA, asthma, gastroesophageal reflux disorder (GERD), and obesity have shared inflammatory pathways; OSA prevalence was also greater among those with asthma patients. The rate of OSA in subjects with asthma at 49.5% [8], in GERD populations was found to be 65% [9]. According to epidemiologic data, the higher prevalence of OSA also occurs in obesity [10].

Currently, continuous positive airway pressure (CPAP) is the optimal therapy for moderate to severe OSA, especially for those without anatomical obstruction [11]. It stabilizes the airway to prevent collapse, improve gas exchange, relieves sleep fragmentation and the chronic intermittent hypoxemia caused by OSA [12] and also improves endothelial cell dysfunction [13]. These improvements benefit OSA symptoms and possibly CV outcomes in some group but not all patients group [14, 15], the effects of CPAP treatment still remain debated.

It is well-known that expansion sphincter pharyngoplasty (ESP) is an effective upper airway surgery choice for the treatment of OSA, successful ESP associated with a reduction in sympathetic activity of the heart, which might be connected with lower incidence of cardiovascular disease [16]. Some studies suggest that CPAP therapy can effectively reduce the risk of cardiovascular (CV) events in OSA patients with previous cardiovascular or cerebrovascular disease [17, 18, 19, 20]. However, recent randomized control trials (RCTs) failed to show the aforementioned benefits [21, 22, 23]. We conducted this meta-analysis of RCTs by synthesizing existing RCTs to investigate the effect of CPAP therapy on prevention of recurrent cardiovascular or cerebral events in OSA patients with history of CV diseases.

## 2. Materials and Methods

### 2.1 Search Strategies

Two authors independently searched PubMed, EMBASE, and the Cochrane Library by using keywords: “Sleep apnea, obstructive”, “Continuous Positive Airway Pressure”, and “randomized controlled trials” from inception through August 2021, without any language restriction. The detail of search strategy is listed in the **Supplementary materials**. Duplicates were removed manually and through EndNote X9 (Thompson ISI Research Soft, Philadelphia, PA, USA). We screened for missing articles by double-checking and retrieving previous meta-analyses (search details in **Supplemental materials**).

#### 2.1.1 Inclusion Criteria

The eligibility criteria: (1) adult patients (aged ≥18 years) with OSA and a history of cardiovascular or cerebrovascular disease; (2) patients who were randomized to either a CPAP or a control group (usual care); (3) outcomes of interest reported; (4) with follow-up period ≥6 months; (5) randomized controlled trials (RCTs).

#### 2.1.2 Exclusion Criteria

(1) Trials performed in child or animals; (2) the control group use other therapy other than usual care; (3) data is not available; (4) follow-up period less than 6 months.

### 2.2 Data Extraction and Management 

The two review authors independently assessed the titles and abstracts, if necessary, browsed the full text and screened the eligible ones based on the inclusion criteria. Any ambiguities and discrepancies were resolved by a third author or a consensus was reached by discussion among all the authors. Two authors independently extracted the data of study baseline characteristics, study design, patients’ demographics, sample size, events (previous/recurrence cardiovascular events), duration of follow-up, time of CPAP use. Risk of bias was evaluated according to the Cochrane Handbook for Systematic Reviews of Interventions (version 5.1.0).

### 2.3 Outcomes

The primary outcome was major adverse cerebral vascular or cardiovascular events (MACCEs), a composite endpoint consisting of cerebrovascular or cardiovascular death, myocardial infarction, stroke (cerebral hemorrhage or infarction). Secondary outcome included cardiovascular events [cardiovascular death, acute myocardial infarction, angina, revascularization, new-onset atrial fibrillation (AF), hospital admission for heart failure (HF), non-fatal stroke, cerebral death and transient ischemic attack (TIA)].

### 2.4 Statistical Analysis 

Analysis was conducted using Review Manager software (version 5.4, The Cochrane Collaboration, Copenhagen, Denmark) and Stata 15.1 (StataCorp, College Station, TX, USA). This study was performed according to the Preferred Reporting Items for Systematic Reviews and Meta-analyses statement (PRISMA) and Cochrane handbook [24, 25]. Heterogeneity was assessed using the chi-squared test with I^2^ statistics among the studies, defined *p* value < 0.1, $I^{2}$ >50% as substantial heterogeneity, if so, he random effect model was used initially, otherwise, the fixed effect model was used [26]. Odds ratio (OR) and 95% confidence interval (CI) were used to compare the efficacy of CPAP on OSA patients with cerebral vascular or cardiovascular diseases. Sensitivity analysis was performed by removing one trial subsequently. Publication bias was checked by funnel plots. Subgroup analyses were performed based on whether the using time is more than 4 hours/night or not.

## 3. Results

### 3.1 Search Results

**Fig. 1.**
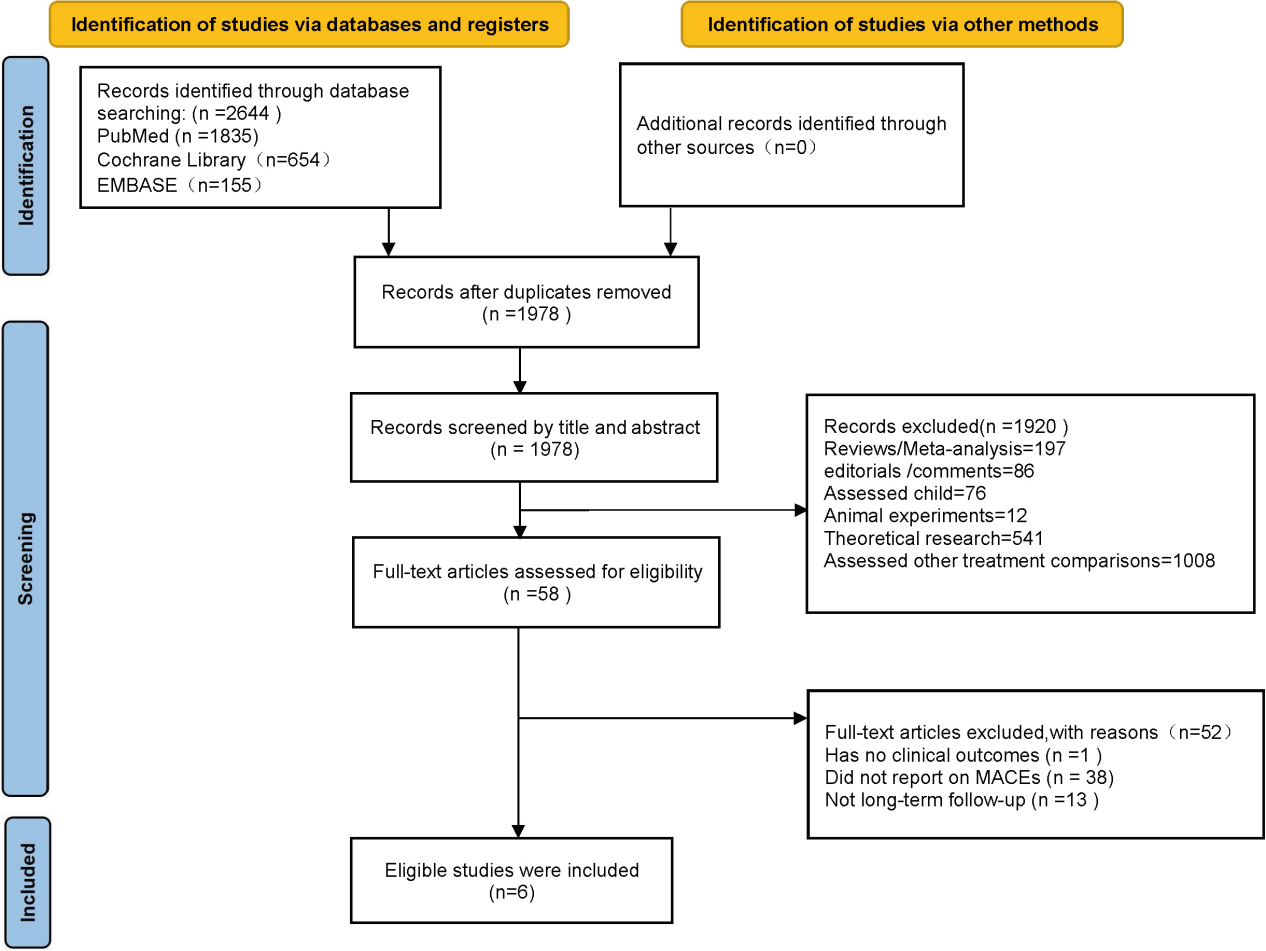
**PRISMA flow chart**. Flow diagram of the studies included in the review and meta-analysis.

As shown in Fig. 1, through electronic retrieval, 2644 references were identified. After deduplication, two independent researchers assessed 1978 studies for eligibility. Of these 1978 studies, the following were excluded: review or meta-analyses (n = 197), editorials/comments (n = 76), animal’s experiments (n = 12), theoretical research (n = 541), and other treatment comparisons (n = 1008). Finally, 58 citations were evaluated by browsing full-text. We subsequently excluded 52 studies, of which, 1 study had no clinical outcomes, and 38 studies reported irrelevant topics, and 13 studies had a follow-up period less than 6 months. Overall, 6 RCTs on relevant topics were included in the final analysis.

### 3.2 Baseline Characteristics 

A total of 4493 participants in 6 studies were eligible for our meta-analysis, of which 2244 (49.9%) patients received CPAP treatment and 2249 (50.1%) were in the usual care group, and the number of participants ranged from 73 to 2687 in each study. The mean age of participants was 62.8 years old (rang between 59.9 and 66.5 years). Males were dominant in each study population (accounting for 69.6% to 86.5%); and obesity was highly prevalent with body mass index (BMI) from 27.5 to 30.2 kg/$m^{2}$. Diagnosis of OSA was identified by either polysomnography (PSG) or cardiorespiratory polygraph (PG) in including studies. Baseline characteristics of included studies are shown in Table 1 (Ref. [21, 22, 23, 27, 28, 29]).

**Table 1.** **The characteristics of the included studies (Ref. [21, 22, 23, 27, 28, 29])**.

Author,Y	Number of participants (CPAP/Control)	Mean time of CPAP useage (hr/night)	Mean age (y) (CPAP/Control)	Male (%) (CPAP/Control)	Mean BMI (kg/$m^{2}$) (CPAP/Control)	Mean AHI (events/h) (CPAP/Control)	Mean ESS (points) (CPAP/Control)	OSA assessment	Coronary artery disease (%)	Diabetes mellitus (%)	Hypertension (%)	Previous stroke (%)
Huang *et al*., 2015 [21]	36/37	4.5	62/62.7	77.8/86.5	27.9/27.5	28.3/28.7	9.3/8.3	PSG	31/38	33/38	NR	NR
Peker *et al*., 2016 [22]	122/122	6.6	65.5/66.5	82/86.1	28.4/28.5	28.3/29.3	5.5/5.5	PG	100/100	27.9/20.5	69/59	NR
Parra *et al*., 2015 [27]	57/69	5.3	63.7/65.5	71.9/69.6	30.2/28.8	≥20/20	8.3/7.3	PG	12.7/17.6	38.2/36.8	60/63	100/100
Traaen *et al*., 2021 [29]	54/54	4.7	63/62	72/80	29.5/29.4	23.1/20.7	8.2/7.5	PG	7/9	7/7	39/43	7/4
Sánchez-de-la-Torre *et al*., 2020 [28]	629/626	2.78	59.9/60.7	84/85	29.6/29.4	36.4/35.5	5.36/5.28	PG	85/85	27/25	56/57	3/3
McEvoy *et al*., 2016 [23]	1346/1341	3.3	61.3/61.2	81.1/80.7	28.8/28.5	29/29.6	7.3/7.5	PG	77/81	30.2/29.4	79/78	53/54

### 3.3 Primary Endpoints

**Fig. 2.**
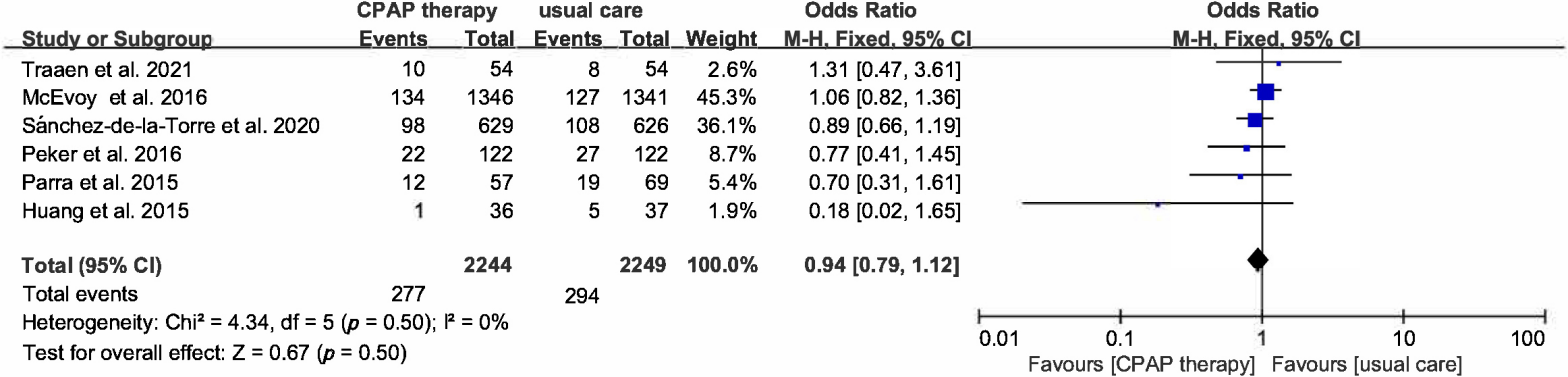
**Forest plot showing effect of CPAP on MACCEs**. Risk of MACCEs in OSA patients treated with CPAP compared to control group.

**Fig. 3.**
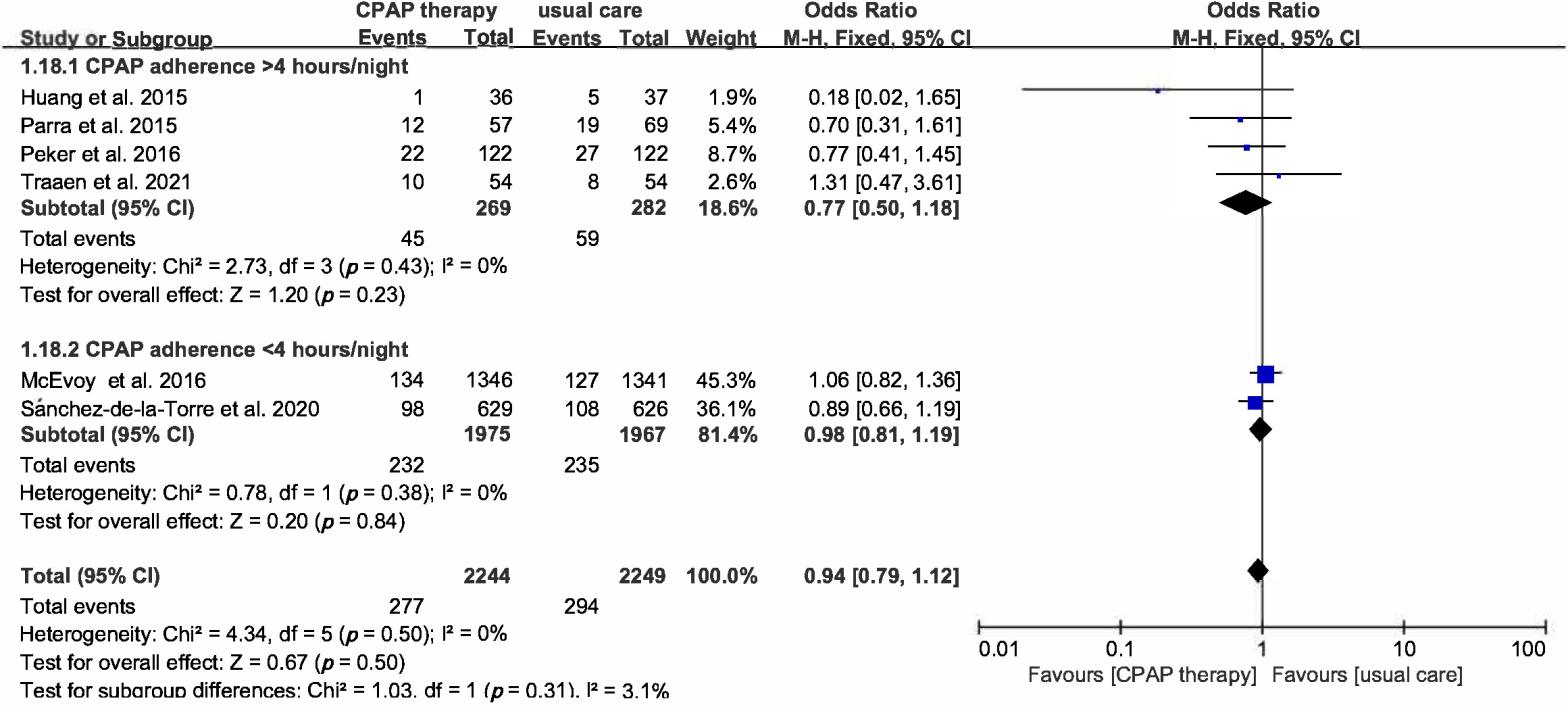
**Forest plot**. Subgroup analysis of the risk of MACCEs in OSA patients treated with CPAP compared to control group based on CPAP usage time.

Six studies [21, 22, 23, 27, 28, 29] reported MACCEs, with mean CPAP adherence 4.53 h/night and mean follow-up duration of 47.83 months. As shown in Fig. 2, Heterogeneity was across trials was not present (*p* = 0.5, $I^{2}$ = 0%), and pooled data from 4493 participants showed a non-significant benefit of CPAP in reducing the risk of MACCEs (OR 0.94, 95% CI [0.79–1.12], *p* = 0.5). Considering that time of CPAP use may potentially have impact on primary outcome, subgroup analysis base on time of CPAP use was performed and there is no difference both in use time >4 hours or <4 hours (OR 0.77, 95% CI [0.5–1.18], *p* = 0.23 vs OR 0.98, 95% CI [0.81–1.19]) (Fig. 3).

### 3.4 Secondary Endpoints

**Fig. 4.**
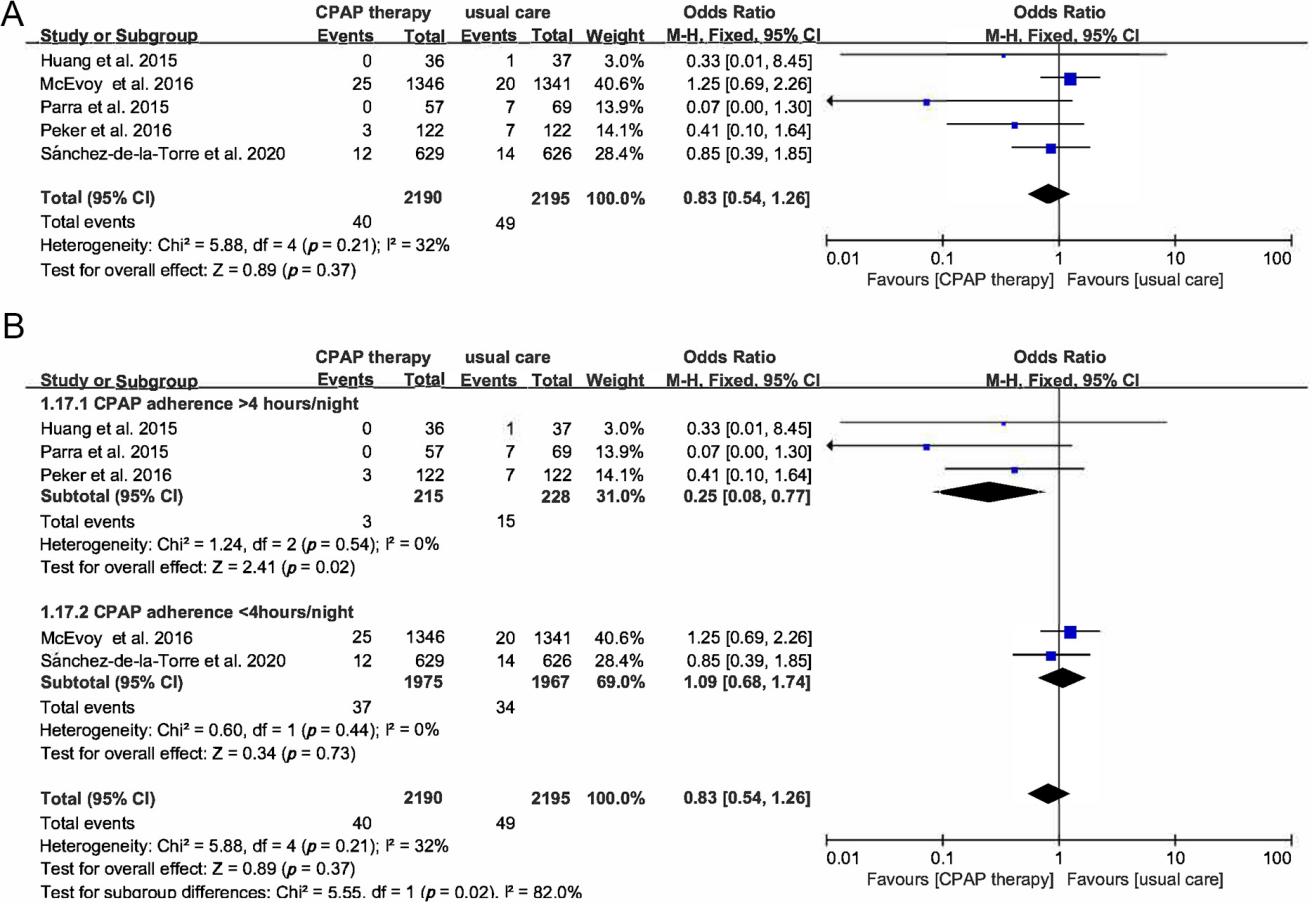
**Forest plot**. (A) Risk of cardiac mortality in OSA patients treated with CPAP compared to control group. (B) Subgroup analysis of the risk of cardiac mortality in OSA patients treated with CPAP compared to control group based on CPAP usage time.

**Fig. 5.**
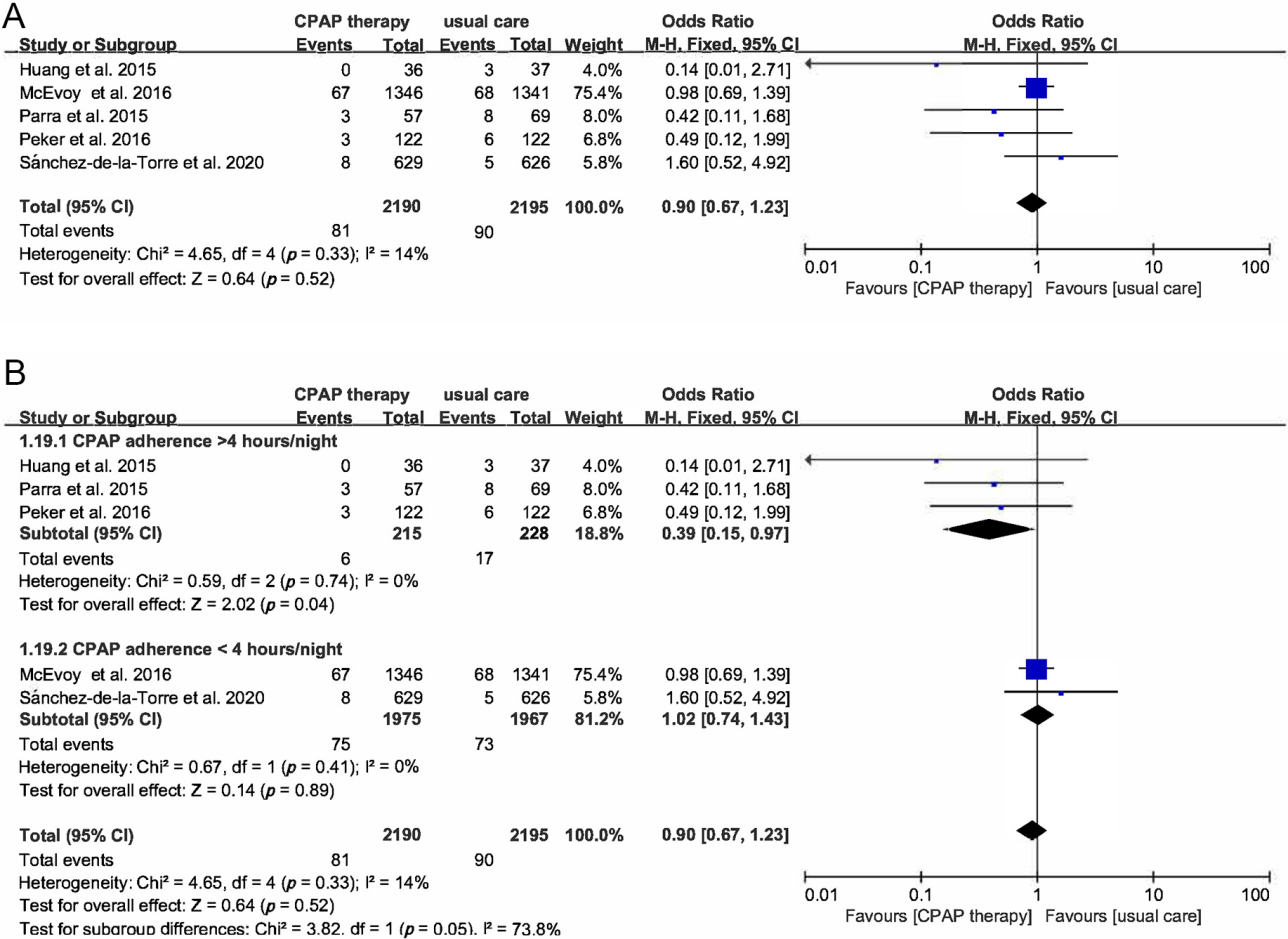
**Forest plot**. (A) Risk of stroke in OSA patients treated with CPAP compared to control group. (B) Subgroup analysis of the risk of stroke in OSA patients treated with CPAP compared to control group based on CPAP usage time.

**Fig. 6.**
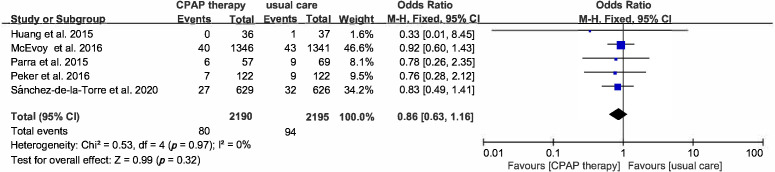
**Forest plot**. Risk of all-cause of death in OSA patients treated with CPAP compared to control group.

There were 5 RCTs of 4385 participants that reporting cardiovascular death, which showed an OR 0.83 (95% CI [0.54–1.26], *p* = 0.37). We further conducted a subgroup analysis of studies which CPAP usage time >4 hours/night showed that the OR of cardiac death was 0.25 (95% CI [0.08–0.77], *p* = 0.02), which significantly reduced the risk of a cardiac death compared with those receiving usual care. A total of 5 studies reported stroke events in 4385 patients. Overall 81 of 2190 (3.7%) participants assigned to the CPAP group suffered stroke compared with 90 of 2195 (4.1%) in the control group (OR 0.9, 95% CI [0.67–1.23], *p* = 0.52). Subgroup analysis based on adequate usage showed the OR of stroke was 0.39 (95% CI [0.15–0.97], *p* = 0.04). All-cause death was evaluated in 5 studies with 4385 patients, the OR of all-cause death was 0.86 (95% CI [0.63–1.16], *p* = 0.32) (Figs. 4A,B,5A,B,6).

### 3.5 Other Cardiovascular Outcomes 

**Fig. 7.**
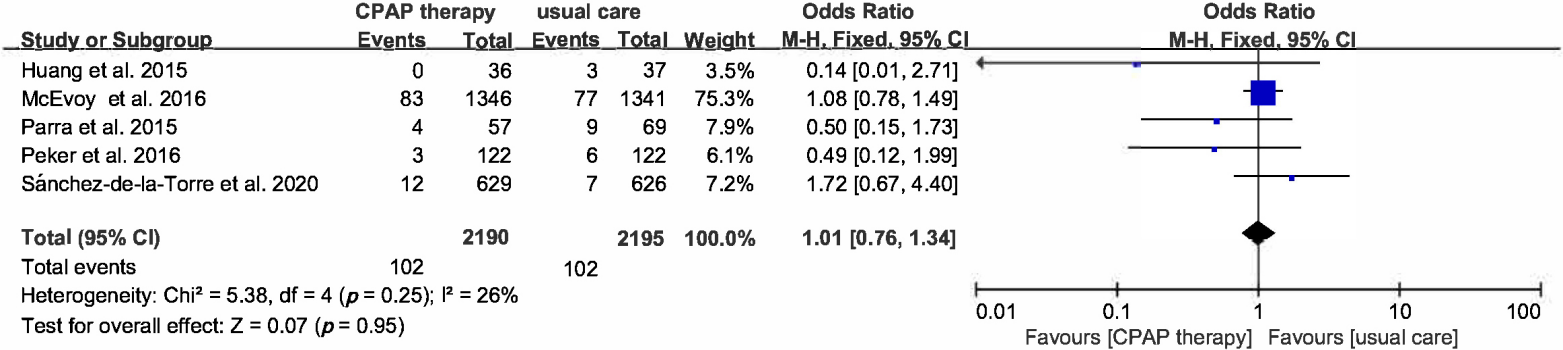
**Forest plot**. Risk of cerebral diseases in OSA patients treated with CPAP compared to control group.

**Fig. 8.**
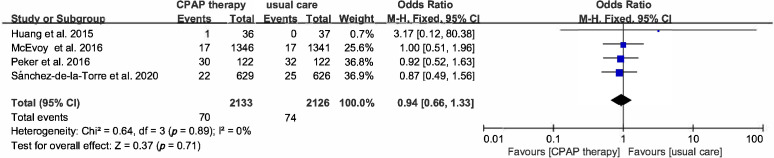
**Forest plot**. Risk of heart failure in OSA patients treated with CPAP compared to control group.

**Fig. 9.**
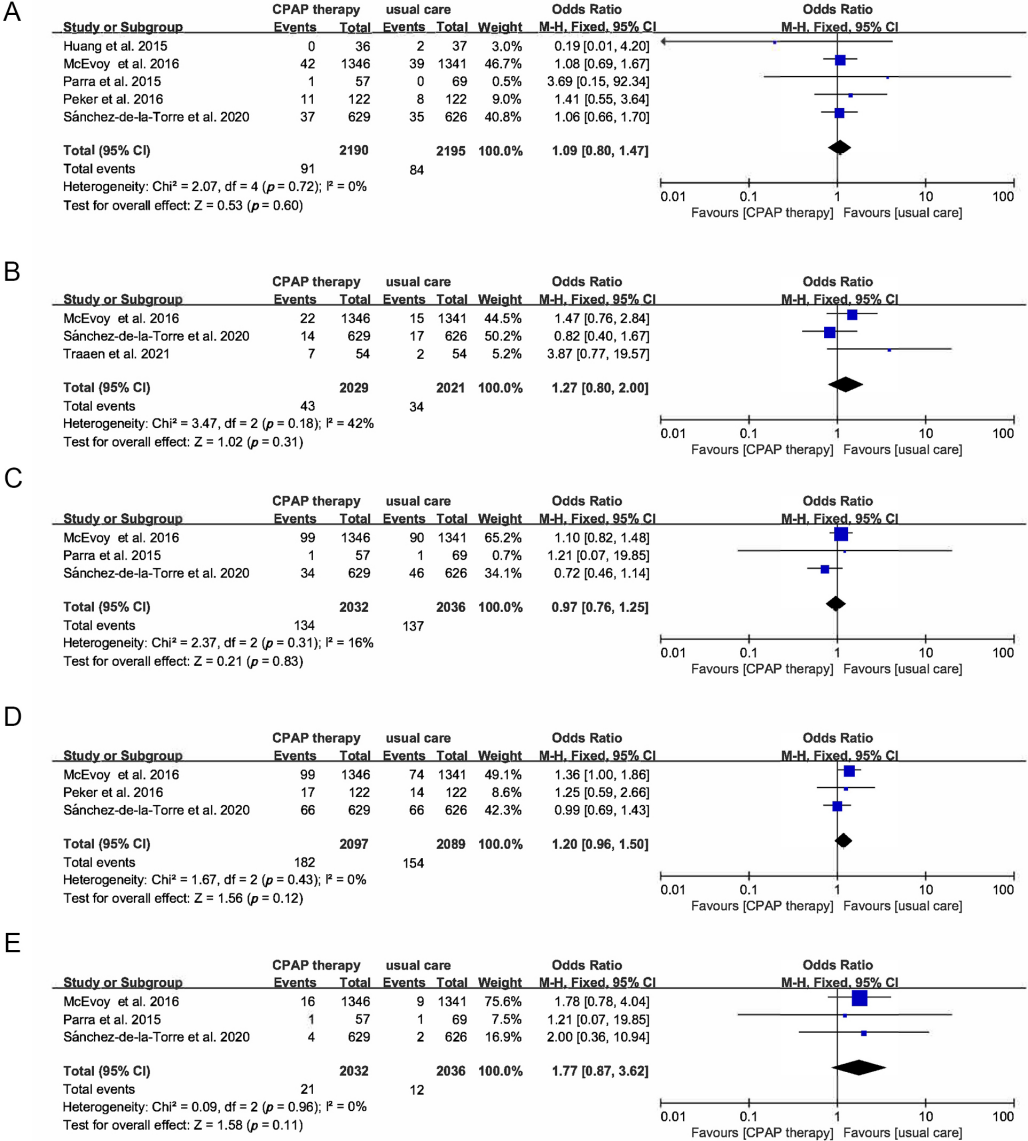
**Forest plot**. (A) Risk of myocardial infarction in OSA patients treated with CPAP compared to control group. (B) Risk of atrial fibrillation in OSA patients treated with CPAP compared to control group. (C) Risk of angina in OSA patients treated with CPAP compared to control group. (D) Risk of revascularization in OSA patients treated with CPAP compared to control group. (E) Risk of transient ischemic attack in OSA patients treated with CPAP compared to control group.

Continuous positive airway pressure therapy was not associated with risk reduction in cerebral disease, HF, MI, AF, angina, revasculation, or TIA. The risk of cerebral disease was reported in 5 RCTs, and pooled data from 4385 participants showed an OR was 1.01 (95% CI [0.76–1.34], *p* = 0.95) (Fig. 7). The effect of CPAP on outcome of HF was assessed in 4 studies, and the OR of HF was 0.94 (95% CI [0.66–1.33], *p* = 0.71) (Fig. 8), MI (OR 1.09, 95% CI [0.8–1.47], *p* = 0.6), atrial fibrillation (OR 1.27, 95% CI [0.8–2], *p* = 0.31), angina (OR 0.97, 95% CI [0.76–1.25], *p* = 0.83), revascularization (OR 1.2, 95% CI [0.96–1.5], *p* = 0.12), TIA (OR 1.77, 95% CI [0.87–3.62], *p* = 0.11) (Fig. 9A–E).

### 3.6 Quality Assessment and Publication Bias 

We used the recommended tool supplied by the Cochrane handbook to assess the risk of bias and the quality of the include trials. Included studies were evaluated as following criteria: whether reported the methods of random sequence generation, allocation concealment, blinding of participants and personnel, the blindness of outcome assessment, incomplete outcome data, selective reporting, and other bias.

**Fig. 10.**
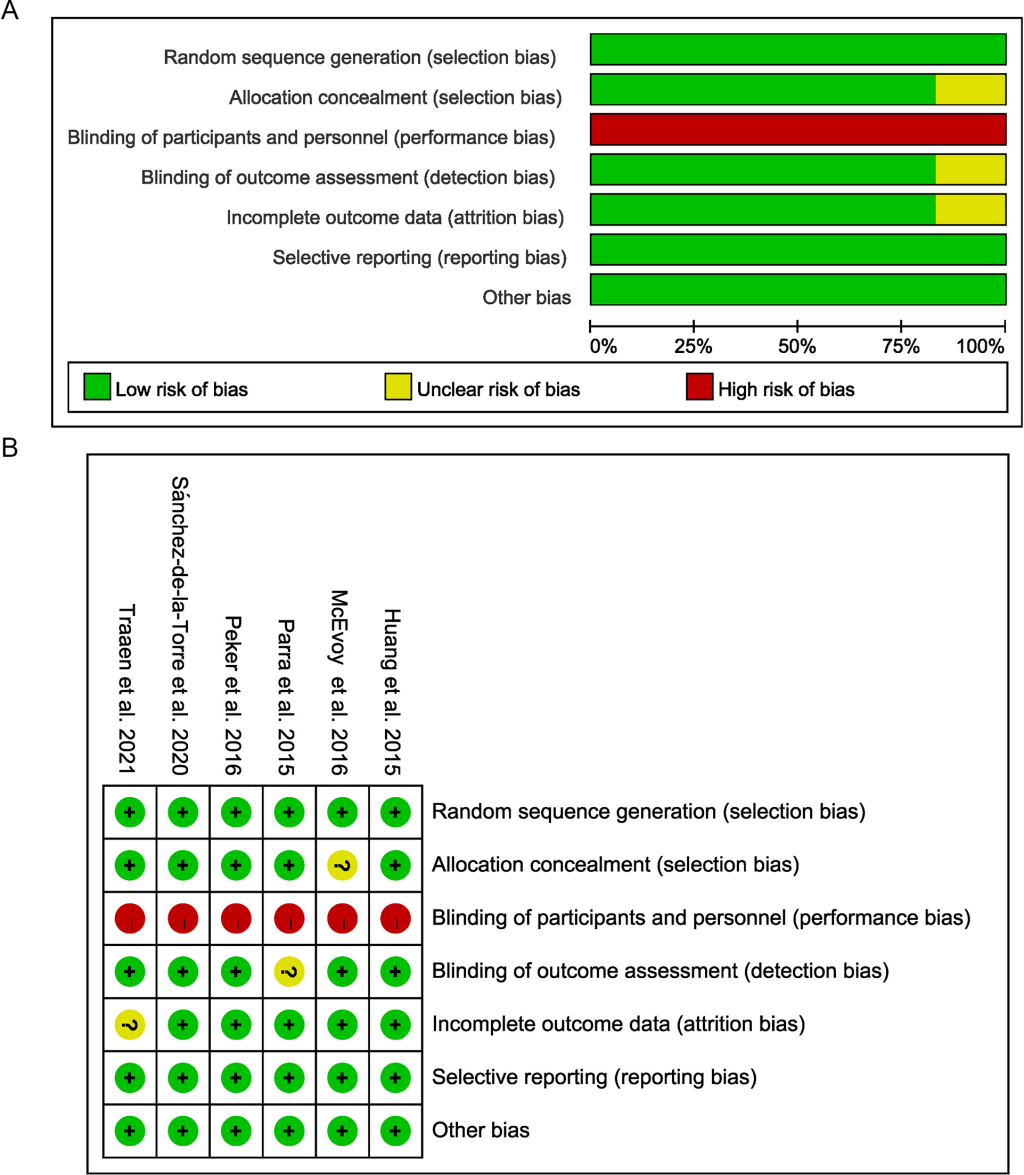
**Risk of bias assessment**. (A) Risk of bias graph: review authors’ judgements about each risk of bias item presented as percentages across all included studies. (B) Risk of bias summary: review authors’ judgements about each risk of bias item for each included study. Green: Low risk of bias; Yellow: unclear risk of Bias and Red: High risk of Bias.

A low risk of bias was seen in several domains, all trials reported adequate random sequence generation and the method of allocation concealment. Allocation concealment was performed by computer-generated randomization software or opaque envelopes. The RCTs compared CPAP therapy with usual care rather than a comparison with sham CPAP. Therefore, there is a high risk of bias in each included study due to lack of blinding of participants and personnel. The assessment of outcome was blinded in all studies that were deemed to have low risk of bias (The details are shown in the Fig. 10A,B and **Supplementary Table 1**). The funnel plot did not show obvious publication bias (**Supplementary Fig. 1**).

### 3.7 Sensitivity Analyses

We conducted a sensitivit**y** analysis to assessed the effect of CPAP adherence on the primary endpoints, by sequentially omitting the trial of low CPAP adherence. It was found that heterogeneity decreased when the SAVE trial [23] was excluded (details are available in the **Supplementary Fig. 2**).

## 4. Discussion

To our knowledge, the present meta-analysis is the first one to focus on assessing the role of CPAP therapy on MACCEs among participants with OSA and previous cardiovascular/cerebrovascular disease. In this systematic review and meta-analysis of current data from 6 RCTs, the key findings were as follows: (1) CPAP therapy failed to significantly reduce the risk of MACCEs or other outcomes, such as myocardial infarction, atrial fibrillation, heart failure, all-cause death, angina, revascularization compared with usual treatment. (2) Subgroup analyses confirmed that adequate use of CPAP (>4 hours) is associated with statistically significant and clinically meaningful improvement in CV mortality and stoke. A previous study has shown that OSA has the most profound adverse effects on stroke and the cerebral circulation [30]. Our findings further demonstrate the importance of adequate CPAP treatment for OSA patients.

A previous review was published by Khan *et al*. [31] that included 7 RCTs (including the SAVE study), which demonstrate that CPAP therapy might reduce MACE when usage time is more than 4 hours/night. Our study is inconsistent with theirs which maybe dues to the fact that this review pooled studies whose participants without previous CV history, and other different interventions such as nocturnal supplemental oxygen, healthy lifestyle and sleep education were used. In our review, five RCTs reported the CV mortality, which showed evidence of an association between CPAP and risk of CV mortality (OR 0.25, 95% CI [0.08–0.77], *p* = 0.02). Labarca *et al*.’s [32] meta-analysis including 8 RCTs studies aimed to assess the benefits of CPAP for secondary prevention in participants with high cardiac risk and previous cardiovascular outcomes, but were unable to show any benefits of CPAP on cardiovascular outcomes. However, they raised concerns about bias, CPAP adherence, and the differing populations included in each RCT which may have reduced the strength of the findings. In patients with a high cardiac risk and previous CV outcomes, pooled data have failed to show an evidence of efficacy of CPAP, and subgroup analysis showed no significant reduction in the risk of cardiovascular mortality and stroke. The results of our subgroup analysis differ from this study which maybe attributed to different participants as our review only included participants with previous cardiovascular events and OSA. Previous studies suggested that CPAP therapy is associated with a slight decrease in blood pressure in non-sleepy patients with OSA [33, 34]. Otherwise, the use of CPAP in OSA with excessive daytime sleepiness, most likely obtain potential cardiovascular benefit [35]. There are only 6 RCT in the final analyses, most studies included patients with non-sleepy or exclude ESS scores over 15 points, thus, further research should collect data on the efficacy of CPAP in pre-specified populations and focus on populations at risk for cardiovascular events. Labarca *et al*.’s [32] review included 3 RCTs which reported primary prevention and others reported secondary prevention of cardiovascular disease. In addition, the SAVE and ISAACC trial occupies a large weight in this analysis, which may have an influence on the results. Besides, Labarca *et al.*’s [32] review included participants who use sham CPAP as control group. As discussed before, there is no true sham CPAP or other placebo for CPAP in long-term studies in OSA patients with cardiovascular/cerebral disease [36]. Furthermore, not everyone can tolerate this therapy because it requires them to sleep with a nasal or full-face mask that is connected by a tube to a machine. Sham positive pressure ventilation may worsen sleep disturbances because of mask coverage and without effective pressure [37]. CPAP adherence is an important factor, and benefits might be underestimated related to lower CPAP compliance. Wang *et al*.’s [38] meta-analysis including 7 observational studies and 2 RCTs studies, which suggested that the benefits of CPAP on prevent subsequent cardiovascular outcomes in patients with cardiovascular disease and OSA, was not verified in RCTs. The result of this study maybe underpowered due to included RCTs studies is not enough.

Although our findings suggest that patient with OSA and a history of cardiovascular disease did not obtain benefits from CPAP therapy in regard to improving either MACCEs or preventing clinically relevant outcomes such as myocardial infraction, a non-significant trend in favor of revealed that CPAP therapy reduce MACEs with adequate time (>4 hours/night) (OR 0.77, 95% CI [0.5–1.18], *p* = 0.23). At the same time, favorable trend with a better compliance time (>4 hours/night) was noticed in cardiac mortality subgroup analysis (OR 0.25, 95% CI [0.08–0.77], *p* = 0.02). Analysis suggested that adequate usage time of CPAP can protect patients from cardiovascular or cerebrovascular death. In other words, CPAP therapy can minimize the harmfulness of OSA complications to a certain extent.

Our findings of CPAP treatment can improve stroke in OSA with previous cardiovascular has clinical implications. On a global scale, stroke ranks as the second cause of death [39]. A growing body of evidence suggests a clear association between OSA and the incidence of stroke [40, 41]. Patients who suffered a stroke have poor quality of life and increased mortality. There are some reasons which could explain this therapy response: when autoregulation is impaired by OSA cycles, cerebral vascular bed fluctuation exposed to OSA-related increase in blood pressure, and chronic intermittent hypoxemia [42]. Therefore, the use of CPAP in OSA patients who suffer from stroke may be necessary. Our findings emphasize the importance of CPAP usage in OSA patients with cerebral diseases, which could potentially prevent adverse cerebrovascular consequences. What’s more, in clinical practice, health care providers should emphasize the importance of CPAP usage time when caring for OSA patients with stroke. Due to intermittent hypoxemia is regarded as a potential contributing factor to the pathogenesis of OSA-related comorbidities, the frequency of hypoxia maybe influents the effect of CPAP, therefore, more studies are needed to explore the differential effects of both short-term and long term hypoxemia about the effect of CPAP treatment.

## 5. Limitation 

There are some limitations in this analysis. First, due to the small number of studies our findings should be interpreted with caution. Second, follow-up times for the included studies were variable (range from 6 months to 84 months), and in one study follow-up was for 6 months only, which was relatively short for a cardiovascular event at follow-up. Lastly, RCTs compared CPAP therapy with usual care rather than a comparison with sham CPAP, which result in a high risk bias of blinding of participants and personnel (A true sham as pointed above is difficult with CPAP). In addition, the studies included in this meta-analysis used different analyses (adjusted, unadjusted, and propensity matched analysis) which could be a source of potential heterogeneity.

## 6. Conclusions

Our meta-analysis suggests that if CPAP is used for more than four hours there is a decrease trend of the incidence of MACCEs, especially, we can observe that increasing CPAP compliance time can have positive effect on cardiac mortality. Considering the risk of bias in the included studies, the usage time of CPAP, the duration of follow-up, and the included population, the credibility of the results may be reduced, and future studies need to improve the quality of CPAP use to further confirm these results.

## Abbreviations

AF, atrial fibrillation; CI, confidence interval; CPAP, continue positive airway pressure; CV, cardiovascular; GERD, gastroesophageal reflux disorder; ESP, expansion sphincter pharyngoplasty; HF, heart failure; MACCEs, Major Adverse Cardiovascular or Cerebrovascular Endpoints; MI, myocardial infarction; OR, odds ratio; OSA, obstructive sleep apnea; PG, cardiorespiratory polygraph; PSG, polysomnography; RCT, randomized controlled trials; TIA, transient ischemic attack.

## Supplementary Material

Supplementary material associated with this article can be found, in the online version, at https://doi.org/10.31083/j.rcm2306195.
