# Supplementary material for: The Effects of Continuous Positive Airway Pressure Therapy for Secondary Cardiovascular Prevention in Patients with Obstructive Sleep Apnoea: A Systematic Review and Meta-Analysis
Source: Rev Cardiovasc Med. 2022 May 27;23(6):195. doi: 10.31083/j.rcm2306195 (PMC11273881; doi:10.31083/j.rcm2306195)
Supplement: Supplementary file 1 [file 2153-8174-23-6-195-s1.zip › 2153-8174-23-6-195-s1.docx]

Supplementary Table 1. Risk of bias of included Randomized Controlled Trials (Ref. [1–6]).

| Source | random sequence generation | allocation concealment | blinding of participants and personnel | blinding of outcome assessment | incomplete outcome data | selective reporting | Other bias |
| --- | --- | --- | --- | --- | --- | --- | --- |
| Huang *et al.* [1] | low | low | high | low | low | low | low |
| Peker *et al.* [2] | low | low | high | low | low | low | low |
| Parra *et al.* [4] | low | low | high | unclear | low | low | low |
| Traaen *et al.* [6] | low | low | high | low | unclear | low | low |
| Sánchez-de-la-Torre *et al.* [5] | low | low | high | low | low | low | low |
| McEvoy *et al.*, [3] | low | unclear | high | low | low | low | low |


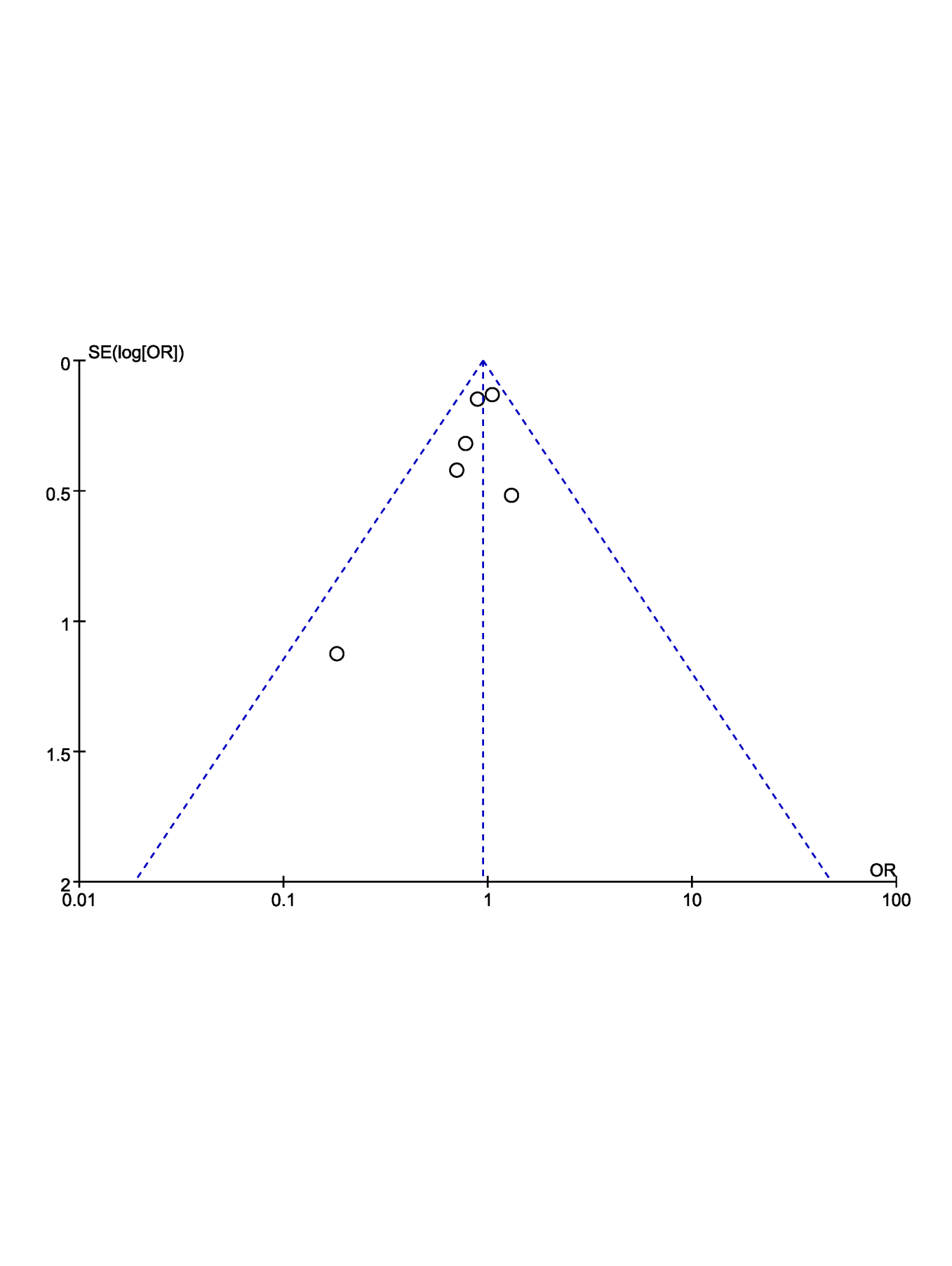


Supplementary Fig. 1. Risk of Bias of Included Randomized Controlled Trials.


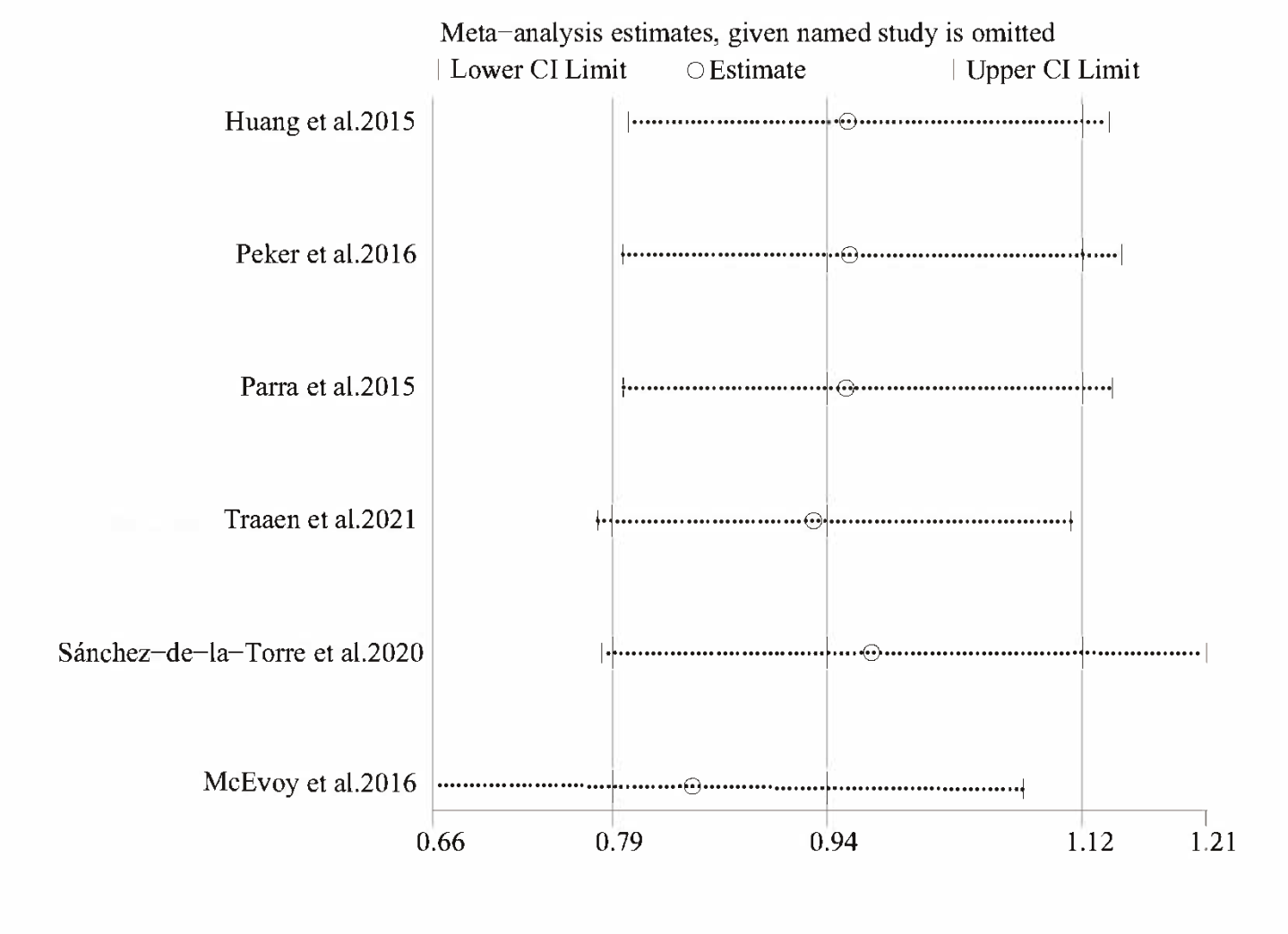


Supplementary Fig. 2. Sensitivity analysis.

References:

[1] Huang Z, Liu Z, Luo Q, Zhao Q, Zhao Z, Ma X, *et al*. Long-Term Effects of Continuous Positive Airway Pressure on Blood Pressure and Prognosis in Hypertensive Patients with Coronary Heart Disease and Obstructive Sleep Apnea: a Randomized Controlled Trial. American Journal of Hypertension. 2015; 28: 300–306.

[2] Peker Y, Glantz H, Eulenburg C, Wegscheider K, Herlitz J, Thunström E. Effect of Positive Airway Pressure on Cardiovascular Outcomes in Coronary Artery Disease Patients with Nonsleepy Obstructive Sleep Apnea. The RICCADSA Randomized Controlled Trial. American Journal of Respiratory and Critical Care Medicine. 2016; 194: 613–620.

[3] McEvoy RD, Antic NA, Heeley E, Luo Y, Ou Q, Zhang X, *et al*. CPAP for Prevention of Cardiovascular Events in Obstructive Sleep Apnea. New England Journal of Medicine. 2016; 375: 919–931.

[4] Parra O, Sánchez-Armengol Á, Capote F, Bonnin M, Arboix A, Campos-Rodríguez F, *et al*. Efficacy of continuous positive airway pressure treatment on 5-year survival in patients with ischaemic stroke and obstructive sleep apnea: a randomized controlled trial. Journal of Sleep Research. 2015; 24: 47–53.

[5] Sánchez-de-la-Torre M, Sánchez-de-la-Torre A, Bertran S, *et al.*, Effect of obstructive sleep apnoea and its treatment with continuous positive airway pressure on the prevalence of cardiovascular events in patients with acute coronary syndrome (ISAACC study): a randomised controlled trial. The Lancet Respiratory Medicine. 2020; 8: 359–367.

[6] Traaen GM, Aakerøy L, Hunt T, Øverland B, Bendz C, Sande LØ, *et al*. Effect of Continuous Positive Airway Pressure on Arrhythmia in Atrial Fibrillation and Sleep Apnea: a Randomized Controlled Trial. American Journal of Respiratory and Critical Care Medicine. 2021; 204: 573–582.
